# Regulation of Hsa‐miR‐4639‐5p expression and its potential role in the pathogenesis of Parkinson's disease

**DOI:** 10.1111/acel.13840

**Published:** 2023-04-26

**Authors:** Lu He, Yimeng Chen, Suzhen Lin, Ruinan Shen, Hong Pan, Yifan Zhou, Ying Wang, Shengdi Chen, Jianqing Ding

**Affiliations:** ^1^ Department of Neurology and Institute of Neurology Ruijin hospital, Shanghai Jiao Tong University School of Medicine Shanghai China; ^2^ Department of Urology, The Third Affiliated Hospital of Soochow University Changzhou Jiangsu China; ^3^ Institute of Aging & Tissue Regeneration Renji Hospital Shanghai Jiao Tong University School of Medicine Shanghai China

**Keywords:** DJ‐1, exosome, histone acetylation, hsa‐miR‐4639‐5p, Parkinson's disease, promoter

## Abstract

Decreased DJ‐1 protein impairs antioxidative activity of neurons and plays an important role in the occurrence of Parkinson's disease (PD). We have previously identified hsa‐miR‐4639‐5p as the post‐transcriptional regulator of DJ‐1. Increased expression of hsa‐miR‐4639‐5p reduced DJ‐1 level and increased oxidative stress leading to neuronal death. Therefore, understanding the detailed mechanisms by which hsa‐miR‐4639‐5p expression is regulated will not only facilitate diagnosis but also inform the pathogenesis of PD. We examined hsa‐miR‐4639‐5 in either the plasma or exosomes derived from the central nervous system (CNS) neurons of PD patients and healthy controls. We showed that CNS‐derived exosomes gave rise to the increased plasma hsa‐miR‐4639‐5p in PD patients, pointing to hsa‐miR‐4639‐5p dysregulation in the brain of PD patients. Using a dual‐luciferase assay and a CRISPR‐Cas9 system, we identified a core promoter of hsa‐miR‐4639 (−560 to −275 upstream the transcriptional starting site) of the gene for myosin regulatory light chain interacting protein. A polymorphism in the core promoter (rs760632 G>A) could enhance hsa‐miR‐4639‐5p expression and increase PD risk. Furthermore, using MethylTarget™ assay, ChIP‐qPCR, and specific inhibitors, we demonstrated that hsa‐miR4639‐5p expression was regulated by HDAC11‐mediated histone acetylation but not DNA methylation/demethylation. Taken together, our study provides evidence that hsa‐miR‐4639‐5p is a potential diagnostic marker and therapeutic target for PD. Interventions targeting hsa‐miR‐4639‐5p might represent a novel therapy to promote healthy aging.

## Graphical abstract

Decreased DJ‐1 impaired antioxidative activity of neurons leading to the occurrence of Parkinson's disease (PD). We have identified hsa‐miR‐4639‐5p as a negative regulator of DJ‐1. Increased miR‐4639‐5p leading to reduced DJ‐1, increased oxidative stress, and neuron death. In PD patients, plasma miR‐4639‐5p was significantly increased. In this study, we found plasma miR‐4639‐5p mainly came from CNS‐derived exosomes and was regulated by HDAC11‐mediated histone acetylation.

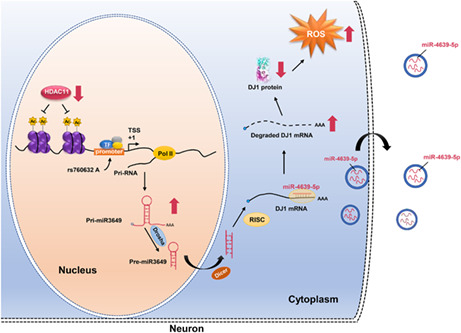

**Abbreviations**
5‐AzaC: 5‐Azacytidine
CGI: the CpG island
ChIP: chromatin immunoprecipitation
CNS: central nervous system
DNMT: DNA methyltransferase
HDAC: histone deacetylase
KO: knock out
L1CAM: neural cell adhesion molecule L1
LDLR: low‐density lipoprotein receptor
LRP8: low‐density lipoprotein receptor‐relatedprotein 8
MPP+: 1‐methyl‐4‐phenylpyridinium
MRLC: myosin regulatory light chain
MYLIP: myosin regulatory light chain interactingprotein
NCAM1: neural celladhesion molecule 1
NTA: nanoparticle tracking analysis
O2‐: superoxide anion
PB: sodium phenylbutyrate
PD: Parkinson's disease
qPCR: quantitative polymerase chain reaction
ROS: reactive oxygen species
SAHA: vorinostat
SB: sodium butyrate
SNP: single nucleotide polymorphism
TEM: transmission electron microscopy
TF: transcriptional factor
TSS: transcriptional start site
VLDLR: very low‐density lipoprotein receptor

## 1 INTRODUCTION

Parkinson's disease (PD) is the second most common neurodegenerative disease which affects approximately 1.7% of the population over 65 years (Ascherio & Schwarzschild, 2016). Due to the increasing life expectancy of the global population, the number of PD patients will further increase as a result of aging, adding a huge burden to the economic and healthcare system (“Global, regional, and national burden of Parkinson's disease, 1990–2016: a systematic analysis for the global burden of disease study 2016,” 2018). Therefore, it is pivotal to understand the pathogenesis at both gene regulation and molecular level to develop both early diagnostic tools and effective treatments for PD.

Oxidative stress plays a leading role in the occurrence and development of PD (Jiang et al., 2016). DJ‐1, an important antioxidative stress protein (Taira et al., 2004), has been implicated in both familial and sporadic PD. In familial PD, deletion or loss of function mutation(s) in the DJ‐1 gene promotes the degradation of DJ‐1 protein by proteasome (Bonifati et al., 2003; Moore et al., 2003). Decreased DJ‐1 protein level weakens its antioxidative stress ability leading to the degeneration of dopaminergic neurons. Similar to familial PD, studies have reported that the level of DJ‐1 in the substantia nigra of sporadic PD patients is also significantly decreased (Kumaran et al., 2009; Nural et al., 2009).

In our previous study of a mouse model of PD, we demonstrated for the first time that DJ‐1 expression was regulated by microRNAs (Xiong et al., 2014). We identified hsa‐miR‐4639‐5p as a key post‐transcriptional regulator for DJ‐1 expression in human; hsa‐miR‐4639‐5p directly targeted the 3′UTR of DJ‐1 to suppress its expression (Chen et al., 2017). The increase in plasma hsa‐miR‐4639‐5p level in PD patients likely contributes to the pathogenesis of PD through downregulating DJ‐1 expression and impairing the antioxidative stress ability of neurons (Chen et al., 2017). MicroRNAs have been reported to be potential biomarkers for diagnosis or prognosis, and even be the therapeutic target for neurodegenerative disease (Ho et al., 2022; Kumar et al., 2013). Because plasma hsa‐miR‐4639‐5p was significantly increased in PD patients compared with controls or essential tremor patients, hsa‐miR‐4639‐5p could serve as a diagnostic marker for PD (Chen et al., 2017). However, the mechanisms by which plasma hsa‐miR‐4639‐5p level is regulated are yet to be defined.

It has been suggested that changes in microRNAs in the blood mirrors changes in microRNAs of the central nervous system (CNS), likely through the exosomes secreted by CNS cells (Zhao & Zlokovic, 2017). However, whether the increased hsa‐miR‐4639‐5p in plasma in PD patients originated from their CNS is still undefined. Careful and thorough investigations of the origin of plasma hsa‐miR‐4639‐5p in PD will not only facilitate the discovery of novel biomarkers but also help to elucidate the exact role of hsa‐miR‐4639‐5p in the pathogenesis of PD.

In the current study, we investigated in PD how the increased plasma hsa‐miR‐4639‐5p was originated and by what mechanism(s) its expression was regulated. We provide evidence that increased hsa‐miR‐4639‐5p in the plasma of PD patients is likely originated from CNS. We further demonstrated that rs760632 G>A polymorphism in the core promoter of hsa‐miR‐4639‐5p could enhance its expression and increase PD risk. Furthermore, we revealed that hsa‐miR4639‐5p expression is regulated by HDAC11‐mediated histone acetylation. The research implies that hsa‐miR‐4639‐5p is a potential diagnostic marker and therapeutic target for PD patients.

## 2 RESULTS

### 2.1 Increased hsa‐miR‐4639‐5p in the plasma of PD patients is likely CNS‐originated

**FIGURE 1:**
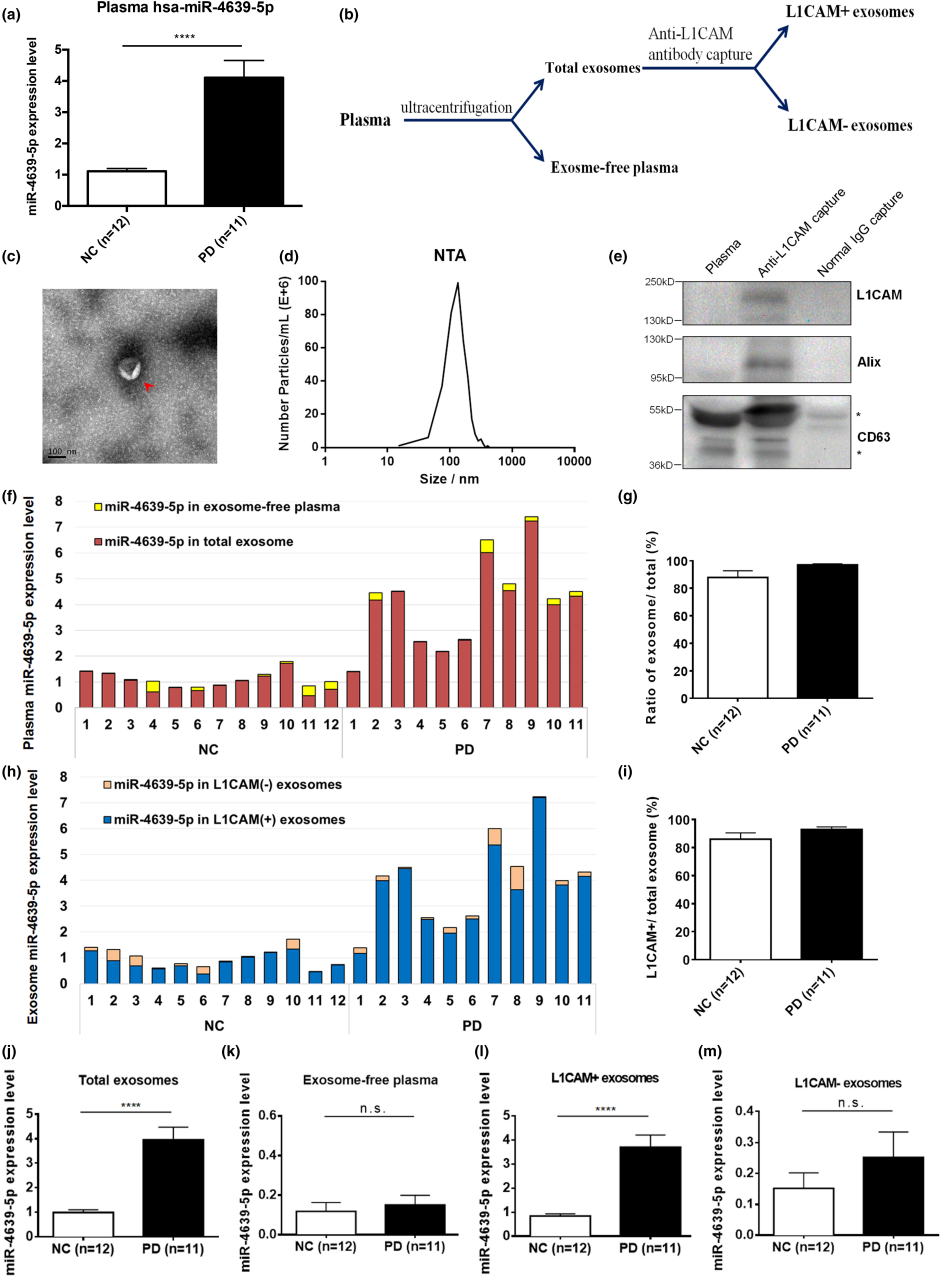
Increased hsa‐miR‐4639‐5p in plasma of PD patients is likely CNS neuron‐derived. (a) qPCR analysis of plasma hsa‐miR‐4639‐5p level in PD patients (*n* = 11) and controls (*n* = 12). (b) The schematic diagram of isolating neuron‐derived exosomes from plasma. (c) Electron microscopy analysis of anti‐L1CAM‐captured plasma exosomes (indicated with red arrows). Scale bar, 100 nm. (d) Size distribution of anti‐L1CAM‐captured plasma exosomes analyzed by nanoparticle tracking system. (e) Western blot examination of the exosome markers Alix, CD63, and L1CAM in anti‐L1CAM captured, plasma (no beads capture) or normal IgG captured components. *Non‐specific bands. (f) The distribution and the concentration of hsa‐miR‐4639‐5p in exosomes and exosome‐free plasma of 11 PD patients and 12 controls. The red part shows the relative miR‐4639‐5p level in exosomes and the yellow part shows the relative miR‐4639‐5p expression level in exosome‐free plasma. The total plasma miR‐4639‐5p level in this figure was calculated with exosome miR‐4639‐5p and exosome‐free plasma miR‐4639‐5p. (g) The average distribution ratio of hsa‐miR‐4639‐5p in exosomes to hsa‐miR‐4639‐5p in plasma. (h) The distribution and the concentration of hsa‐miR‐4639‐5p in neuron‐derived exosomes and non‐neuron‐derived exosomes. The blue part showed the relative hsa‐miR‐4639‐5p level in L1CAM (+) exosomes and the pink part showed the relative hsa‐miR‐4639‐5p expression level in L1CAM (−) exosomes. The total exosome miR‐4639‐5p level in this figure was calculated with L1CAM (+) exosome miR‐4639‐5p and L1CAM (−) exosome miR‐4639‐5p. (i) The average distribution ratio of hsa‐miR‐4639‐5p in L1CAM‐containing exosomes to hsa‐miR‐4639‐5p in total exosomes. The concentration of hsa‐miR‐4639‐5p in total exosomes (j), exosome‐free plasma (k), L1CAM‐containing exosomes (l) or non‐L1CAM‐containing exosomes (m) separated from plasma samples of PD patients and controls.

Neural cell adhesion molecule L1 (L1CAM) is a fundamental member of a cell adhesion molecule subfamily, which is mainly expressed on the surface membrane of neurons (Schmid & Maness, 2008). LICAM is thus considered to be a bona fide surface marker for exosomes derived from the CNS neurons. To isolate those plasma exosomes likely derived from CNS, L1CAM‐containing exosomes from the plasma of 11 PD patients and 12 normal controls were immune‐captured following an established protocol (Shi et al., 2014; Figure 1a,b). Enrichment of exosome markers such as Alix, CD63, and L1CAM in the anti‐L1CAM captured exosomes was verified by Western blot analysis, demonstrating successful isolation of L1CAM‐containing exosomes derived from CNS (Figure 1e). Transmission electron microscopy (TEM) and nanoparticle tracking analysis (NTA) were used to examine the quality and size distribution of the anti‐L1CAM captured exosomes (Figure 1c,d). The morphological characteristics of the L1CAM‐containing exosomes are similar to the exosomes reported before, with a diameter between 30 and 200 nm (Pegtel & Gould, 2019).

We then used qPCR to measure the hsa‐miR‐4639‐5p level in each separated sample (Figure 1b). We found that hsa‐miR‐4639‐5p was predominantly presented in the exosome fractions, but rarely in the plasma that was devoid of the exosome, in samples from both PD patients and normal controls (Figure 1f,g). Furthermore, the L1CAM‐containing exosomes contained the highest levels of hsa‐miR‐4639‐5p (Figure 1h,i). In PD patients, hsa‐miR‐4639‐5p was significantly increased in L1CAM‐containing exosomes, but not in non‐L1CAM‐containing exosomes or exosome‐free plasma (Figure 1j–m). These results suggested that the increased plasma hsa‐miR‐4639‐5p in PD was likely originated from the CNS‐derived exosomes.

To further confirm the result, we isolated CNS neuron‐derived exosomes with neural cell adhesion molecule 1 (NCAM1), another neuron surface marker. As in the L1CAM‐containing exosomes, plasma hsa‐miR‐4639‐5p was mainly present in the NCAM1‐containing exosomes. In PD patients, hsa‐miR‐4639‐5p was significantly increased in the NCAM1‐containing exosomes, but not in the non‐neuron‐derived exosomes (Figure S1). Taken together, our results demonstrate that the abnormally increased hsa‐miR‐4639‐5p found in the plasma of PD was likely via exosomes derived from the CNS neurons.

### 2.2 Hsa‐miR‐4639‐5p shares the promoter with its host gene MYLIP

**FIGURE 2:**
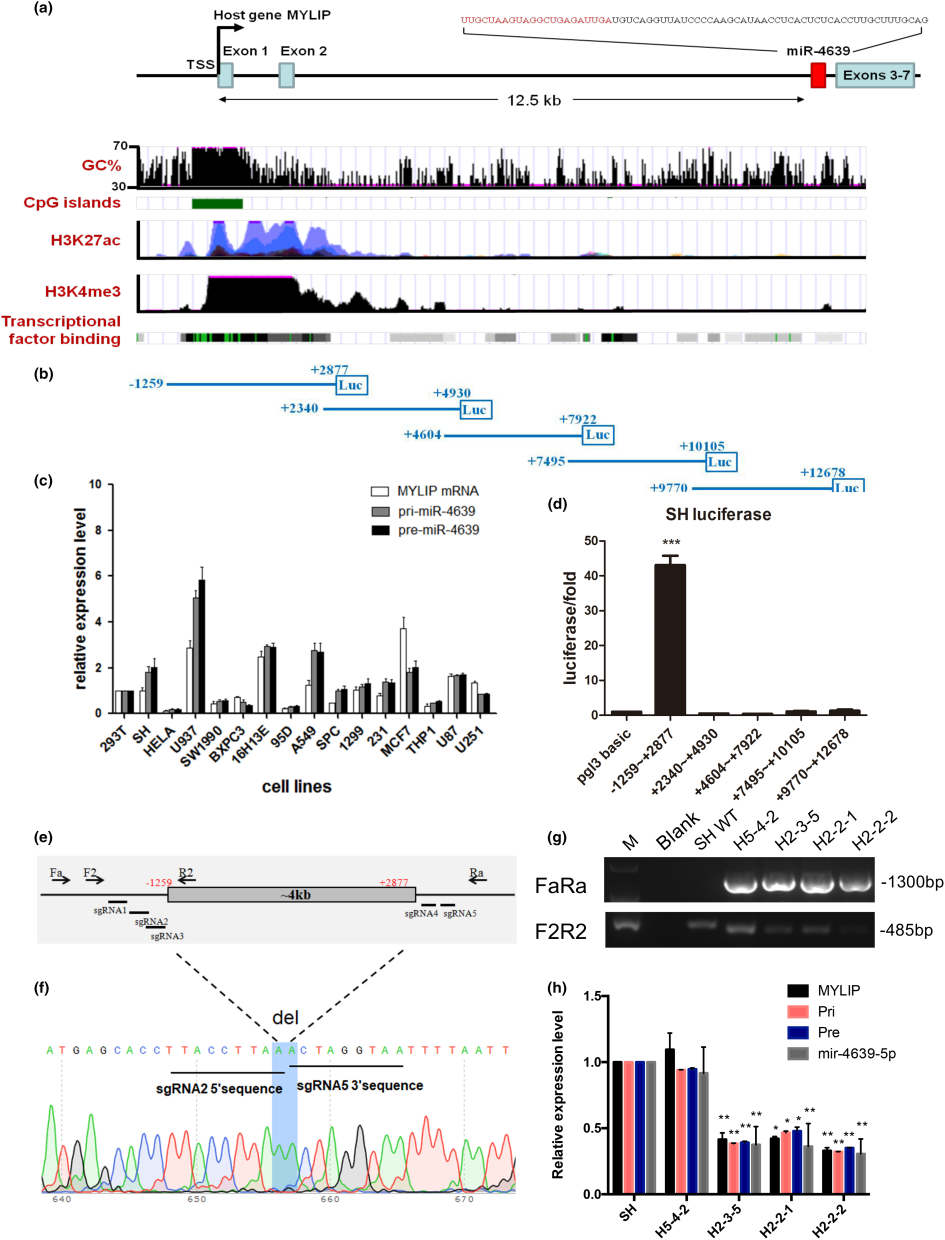
Hsa‐miR‐4639‐5p shares the promoter with its host gene MYLIP. (a) Diagram of gene position of hsa‐miR‐4639 and MYLIP from UCSC database, with ENCODE tracks for CpG islands, histone marks, and TF‐binding signals. The pre‐miR‐4639 sequence is shown, with the mature miR‐4639‐5p sequence highlighted in red. TSS: transcriptional start site. (b) Schematic representation of constructed dual‐luciferase reporter plasmids containing different regions of the miR‐4639 upstream sequence. (c) Expression profile of pri‐miR‐4639, pre‐miR‐4639, and host gene MYLIP in different human cell lines originated from different human tissues. (d) Dual‐luciferase reporter assay examined promoter activity of the fragments shown in (b). The normalized luciferase activity, as the mean + SEM of at least three independent experiments is shown. (e) The schematic diagram of genome editing (knocking out the −1259~+2877 fragment) with CRISPR‐Cas9. Gene position of gRNAs (sgRNA 1–6) and genotyping primers (Fa + Ra and F2 + R2) are shown. (f) Sequencing result of the successful KO cell line H2‐2‐2 is shown. (g) Genotyping results for examining the knockout efficiency of the −1259~+2877 fragment from the genome with CRISPR‐Cas9. (M: marker, Blank: the negative control of PCR with no DNA template. SH WT: the blank group (SH‐SY5Y cells transfected with Cas9 blank plasmid (without sgRNAs) followed by single‐cell selection), H5‐4‐2, H2‐3‐5, H2‐2‐1 and H2‐2‐2: single cell‐derived cell lines following sgRNA‐Cas9 plasmid transfection and screening.) (h) The expression profile of endogenous pri‐miR‐4639, pre‐miR‐4639, miR‐4639‐5p, and MYLIP in cell lines in (g). The experiment was repeated 3 times independently. Each cell group is compared with the blank SH group using two‐way ANOVA. *p < 0.05, **p < 0.01.

To investigate the possible regulatory mechanism resulted in abnormally elevated hsa‐miR‐4639‐5p levels in PD patients, we next examined regulating mechanism of hsa‐miR‐4639‐5p transcription. One of the challenges is that the promoter region and upstream regulators of hsa‐miR‐4639‐5p have not yet been reported. The human hsa‐miR‐4639 is located on Chr6: 16141556–16141624 [+] (GRCh38), which is in the intron region of MYLIP (myosin regulatory light chain interacting protein) gene. UCSC database analysis suggested a predicted promoter region of 12.5‐kb upstream the sequence of hsa‐miR‐4639‐5p. It is located near the transcriptional start site (TSS) of the MYLIP gene, with a high GC%, enriched H3K27ac, H3K4me3 signals, and strong transcriptional factors (TFs) binding signals (Figure 2a). The position signature suggests that the microRNA may be transcribed together with its host gene MYLIP (Kim et al., 2009). The qPCR assays conducted using human cell lines originated from different tissues showed the expression profile of endogenous MYLIP mRNA, pri‐miR‐4639 and pre‐miR‐4639 were highly consistent (Figure 2c), confirming that these three mRNA species are likely co‐transcribed.

To define the promoter of hsa‐miR‐4639, a series of luciferase reporter plasmids containing different regions of the upstream sequence of miR‐4639 were constructed (Figure 2b). Among which only the fragment of −1259~+2877 showed high transcriptional activity (about 40 times than that of the empty plasmid; Figure 2d). We further knocked out (KO) the fragment of −1259~+2877 in SH‐SY5Y cells using a CRISPR‐Cas9 gene‐editing system. After a series of screening and identification, we established three KO cell lines (H2‐3‐5, H2‐2‐1, and H2‐2‐2; Figure 2e–g). These cell lines were further confirmed by qPCR. Our results showed that expression of the endogenous miR‐4639 precursors, mature miR‐4639‐5p, and MYLIP mRNA were all significantly reduced by 60%–70% in these cell lines (Figure 2h). These results further proved that hsa‐miR‐4639‐5p shared the promoter with the MYLIP gene.

### 2.3 Identification of the core promoter sequence of the hsa‐miR‐4639‐5p

**FIGURE 3:**
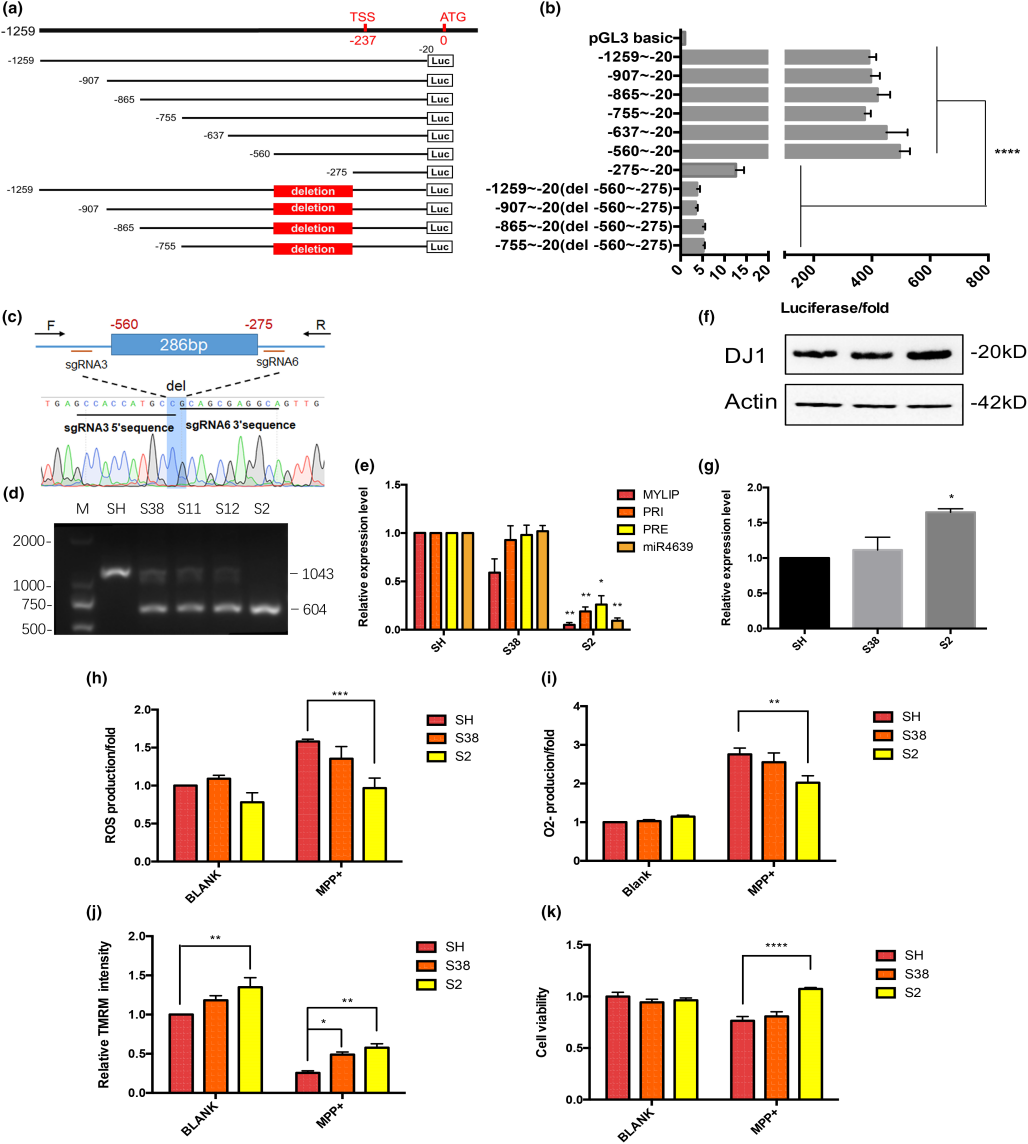
Core promoter identification of the hsa‐miR‐4639‐5p. (a) Schematic representation of dual‐luciferase reporter constructs containing different fragments of −1259 to −20 upstream of the translation starting site (ATG). TSS: transcriptional start site. (b) Dual‐luciferase assay in human SH‐SY5Y cells transfected with luciferase reporter vectors listed in (a). The normalized luciferase activity, as the mean + SEM of at least three independent experiments, is shown. (c) The schematic diagram of genome editing (knocking out the −560 to −275 fragment) with CRISPR‐Cas9. Gene position of sgRNAs (sgRNA3 and sgRNA6) and genotyping primers (F and R) and sequencing result of the successful KO cell line S2 are shown. (d) Genotyping results for identifying the successfully −560 to −275 fragment knockout cell lines. M: marker, SH: the blank group (SH‐SY5Y cells transfected with Cas9 blank plasmid (without sgRNAs) followed by single‐cell selection), S38, S11, S12, and S2: single cell‐derived cell lines following sgRNA‐Cas9 plasmid transfected and screened. (e) The expression profile of endogenous pri‐miR‐4639, pre‐miR‐4639, miR‐4639‐5p, and MYLIP in the blank cell line SH, not successful KO cell line S38 and the KO cell line S2. (f) Western blot results of DJ‐1 protein level in hsa‐miR‐4639‐5p core promoter KO cell line S2 and controls. (g) Quantification plots of protein level in (f). CM‐H2DCFDA assay to check the ROS production (h), DHE assay to measure the O2‐production (i), TMRM assay to examine mitochondrial membrane potential (j), and CCK‐8 assay to examine cell viability (k) of S2 and controls treated with MPP+ or not. Each experiment was repeated three times independently. Each cell group is compared with the blank SH group using two‐way ANOVA. *p < 0.05, **p < 0.01, ***p < 0.001, ****p < 0.0001.

When compared to other regions upstream of miR‐4639‐5p, the −1259 to +2877 fragment, in particular, showed the highest transcriptional activity, suggesting that this region contains the promoter for miR‐4639‐5p. To identify the core promoter region of hsa‐miR‐4639‐5p, a series of 5′ truncated sequences with the 3′ end fixed at the +2877 locus were fused to the luciferase in plasmid expression vector (Figure S2). The luciferase activity assay showed that the long fragment downstream of the translation starting site ATG (−20~+2877) had no contribution to the transcriptional activity. We then focused on the −1259 to −20 region upstream of the ATG. As shown in Figure 3a,b, while the −275 to −20 fragment showed almost no transcriptional activity, the 5′ end extending to −560 increased the luciferase activity by 40‐fold consistently. On the contrary, deletion of the −560 to −275 fragment from the construct resulted in a sharp decrease in the transcriptional activity. We then used CRISPR‐Cas9 to knock out the −560 to −275 fragment in SH‐SY5Y cells and used qPCR to measure the transcription activity in one of the KO cell lines (the S2 cell line; Figure 3c,d). The results showed the precursor of hsa‐miR‐4639, mature hsa‐miR‐4639‐5p, and MYLIP mRNA were all significantly decreased in this KO cell line (Figure 3e). These data are evidence that the −560 to −275 fragment is the core promoter for hsa‐miR‐4639‐5p.

We then measured the DJ‐1 protein level in SH‐SY5Y cells with the core promoter region for hsa‐miR‐4639‐5p deleted. Our results showed that DJ‐1 protein level was significantly increased in the S2 cell line (Figure 3f,g). To define the increase in DJ‐1 was specifically resulted from the deletion of hsa‐miR‐4639‐5p core promoter region, we examined the impact of knocking out MYLIP. MYLIP is an E3 ubiquitin‐protein ligase that mediates the ubiquitination and proteasomal degradation of myosin regulatory light chain (MRLC), low‐density lipoprotein receptor (LDLR), very low‐density lipoprotein receptor (VLDLR), and low‐density lipoprotein receptor‐related protein 8 (LRP8). MYLIP is not considered to be associated with DJ‐1, oxidative stress, or Parkinson's disease (Lindholm et al., 2009). We found that the alteration of MYLIP expression did not impact the DJ‐1 protein level (Figure S3).

In the pathogenesis of PD, 1‐methyl‐4‐phenylpyridinium (MPP^+^) induced oxidative insult is thought to be a significant environmental cause of dopaminergic neuron death, an effect that can be counter‐acted by DJ‐1. To assess the oxidative stress level in the core promoter KO cells, we measured ROS (the overall reactive oxygen species mainly consists of superoxide anion and hydrogen peroxide) and superoxide anion ($O_{2}^{-}$). The results indicated that these cells treated with MPP^+^ produced less ROS and $O_{2}^{-}$ and showed less mitochondrial damage than control cells treated with MPP^+^ (Figure 3h–j). In addition, the core promoter KO cells were more resistant to MPP^+^‐induced cell damage (Figure 3k). The results suggest that knocking out the core promoter region of hsa‐miR‐4639‐5p could act by increasing DJ‐1 protein to enhance cell resistance to MPP^+^‐induced oxidative insult, thus improve cell viability.

### 2.4 The rs760632 polymorphism in the core promoter region influence the expression of hsa‐miR‐4639‐5p

Polymorphic sites in the core promoter region may influence transcriptional activity by affecting the binding and regulatory ability of transcription factors to the promoter. Whether polymorphisms in the hsa‐miR‐4639‐5p promoter affect the transcriptional activity that is correlated to the occurrence of PD is unknown. Thus, we sequenced the hsa‐miR‐4639‐5p promoter region in 308 PD patients and 357 normal controls (Figure S4a). Two single nucleotide polymorphism (SNP) sites were detected in or near the core promoter region, of which rs760632 was located inside the core promoter (Figure S4b), and the G>A variation of this site increased the risk of PD (Figure S4c). We used the luciferase assay to explore whether this variant impacted the transcriptional activity of the promoter. The results showed that the rs760632 G>A variation enhanced the transcriptional activity of hsa‐miR‐4639‐5p promoter (Figure S4d). To further verify the effects of the SNP variant on hsa‐miR‐4639‐5p expression, the relationship between the genotype of rs760632 and the expression of hsa‐miR‐4639‐5p in plasma was further analyzed in PD patients and normal controls. In both populations (Figure S4f,g), person bearing the rs760632 G>A variant showed a higher plasma hsa‐miR‐4639‐5p level than PD patients who did not harbor this SNP. Again, PD patients had higher hsa‐miR‐4639‐5p level than that of the controls (Figure S4e). Our data have demonstrated that the polymorphisms in the core prompter region may partially account for the abnormal upregulation of hsa‐miR‐4639‐5p in PD, although other regulatory mechanisms of hsa‐miR‐4639‐5p such as epigenetic modification may also contribute.

### 2.5 Downregulation of DNA methylation in the promoter region does not change transcriptional activity of hsa‐miR‐4639‐5p

**FIGURE 4:**
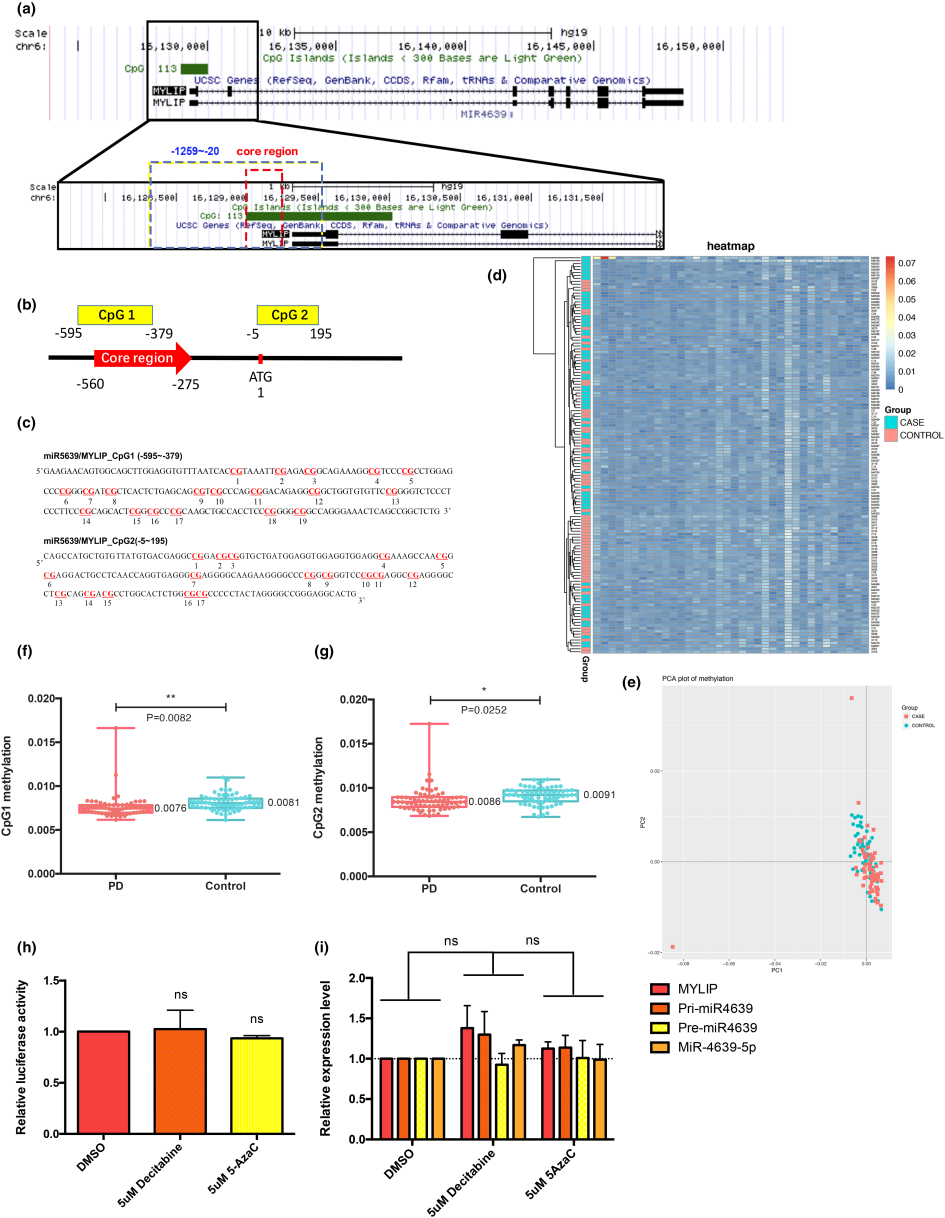
DNA methylation is not involved in the regulation of hsa‐miR‐4639‐5p. (a) Diagram of MYLIP and miR4639 from UCSC genome browser, illustrating location of identified promoter regions (core region shown in red and −1259~−20 shown in blue). (b) Schematic view of the relative positions of the CpG islands investigated in the study. (c) Sequence of the CpG1 island existing in the core promoter region and the CpG2 island adjacent to the start codon ATG. (d) Heat map of DNA methylation data of PD patients (shown as CASE) and controls (shown as CONTROL). Each cell represents the relative methylation level of the CpG locus of the corresponding row sample, and reflects the methylation level change with a color gradient. The tree diagram on the left side of the figure systematically describes the similarity of the sample methylation level. (e) Principal component analysis (PCA) plot of the DNA methylation data of PD patients and controls. Comparison of the average methylation degree of CpG1 (f) and CpG2 (g) between PD patients and normal control. (h) Dual‐luciferase assay results assessing the effect of DNMT inhibitors Decitabine and 5‐AzaC to the transcriptional activity of hsa‐miR‐4639‐5p. (i) The expression level of hsa‐miR‐4639‐5p in the presence of DNMT inhibitors. Student's unpaired *t* test, one‐way ANOVA, bars are mean ± SEM. *p < 0.05, **p < 0.01, ***p < 0.001, ****p < 0.0001, ns *p* > 0.05, compared with the DMSO group.

Epigenetic modifications such as DNA methylation and histone acetylation in the promoter region often regulate microRNA expression. Approximately 50% of microRNA expression are associated with methylation of the CpG islands (CGIs; Wang et al., 2013). Generally, methylated CGIs leads to silencing of the associated promoter (Deaton & Bird, 2011). To determine whether there is an alteration on DNA methylation level of hsa‐miR‐4639‐5p promoter in PD, predicted CGIs (Figure 4a–c) and the 2 CpG regions adjacent to the core promoter region were selected and sequenced using MethylTarget™ assay in 50 PD patients and 50 gender‐age matched controls. As shown in Figure 4d, both in PDs and controls, the DNA methylation level of promoter was very low. There was only an 0.1% increase in the methylation level in the controls compared with PD samples in the total methylation percentages of both CpG1 and CpG2 (CpG1: 0.76% methylated in PD vs. 0.81% in controls, CpG2: 0.86% methylated in PD vs. 0.91% in controls, Figure 4f,g). Although there was a statistical difference between PD and control, such a low level of DNA methylation was considered to have little influence on the transcriptional activity (Deaton & Bird, 2011).

To further confirm whether DNA methylation participates in the regulation of hsa‐miR‐4639‐5p, DNA methyltransferase (DNMT) inhibitors Decitabine and 5‐Azacytidine (5‐AzaC) were used to treat the SH‐SY5Y cells. As shown in Figure 4h,i, neither the transcriptional activity nor the expression level of hsa‐miR‐4639‐5p was affected by DNMT inhibitors, further indicating that DNA methylation was likely not involved in the regulation of hsa‐miR‐4639‐5p.

### 2.6 Histone acetylation regulates the expression of hsa‐miR‐4639‐5p

**FIGURE 5:**
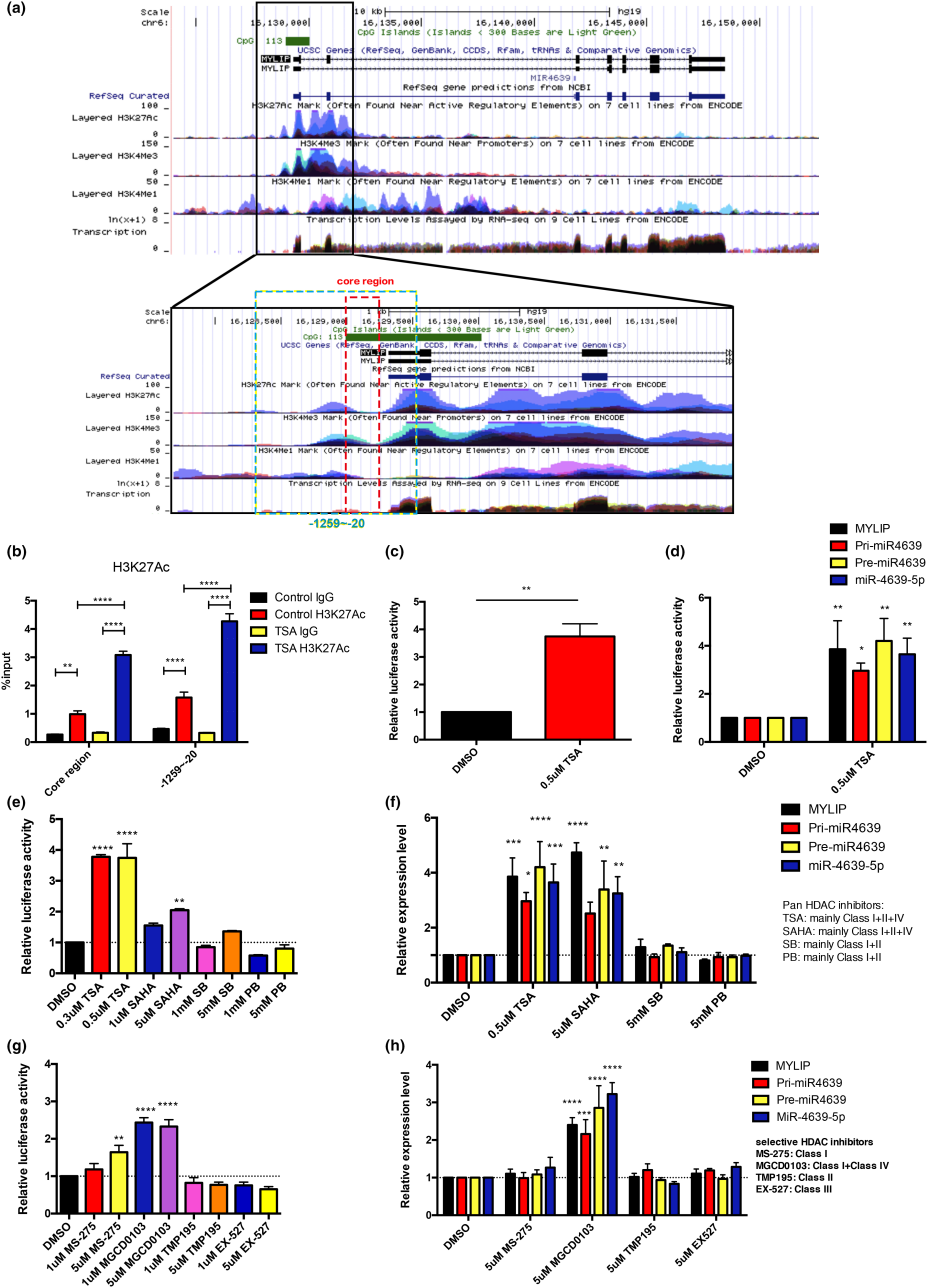
Class IV HDACs might play the most important role in the regulation of hsa‐miR‐4639‐5p. (a) Diagram of MYLIP and miR4639 from UCSC genome browser, demonstrating ENCODE tracks for histone marks at the promoter region. The location of amplified regions in (b) was shown (core region shown in red and −1259~−20 shown in blue). (b) ChIP assays for the two promoter regions (core region and −1259~−20) with analysis using qPCR for enrichment of H3K27ac normalized to the % input. (c) Effects of the TSA treatment on the transcriptional activity of core promoter region of the hsa‐miR‐4639‐5p. (d) Effects of the TSA treatment on the expression of MYLIP, pri‐, pre and mature miR‐4639‐5p. Effects of pan (e) or selective (g) HDAC inhibitors on the transcriptional activity of core promoter region of the hsa‐miR‐4639‐5p. Effects of pan (f) or selective (h) HDAC inhibitors on the expression of MYLIP, pri‐, pre‐, and mature miR‐4639‐5p. Student unpaired t test and two‐way ANOVA with “Dunnett's multiple comparisons test” are used, bars are mean ± SEM. *p < 0.05, **p < 0.01, ***p < 0.001, ****p < 0.0001, compared with the DMSO group.

In addition to DNA methylation, histone acetylation is also one of the most common epigenetic modifications that regulate gene expression. To determine whether histone acetylation was involved in the regulation of hsa‐miR‐4639‐5p, SH‐SY5Y cells were cultured with the pan histone deacetylase (HDAC) inhibitor TSA. We performed chromatin immunoprecipitation (ChIP) and quantitative PCR (qPCR) assays. Our results demonstrated a significant enrichment of the transcriptional activation associated histone marker H3K27Ac at the promoter region of hsa‐miR‐4639‐5p after TSA treatment (Figure 5b). The results of luciferase assay and qPCR assay showed the increase in transcriptional activity and expression level of pri‐, pre‐, and hsa‐miR‐4639‐5p (Figure 5c,d). Our data are evidence that histone acylation is an important regulatory mechanism for increased expression of hsa‐miR‐4639‐5p.

To further define the effect of histone acetylation on the expression of hsa‐miR‐4639‐5p, we tested other pan‐HDAC inhibitors SAHA (Vorinostat), SB (Sodium butyrate), and PB (Sodium phenylbutyrate). TSA and SAHA showed a significant stimulating effect while SB and PB had no effect on the transcriptional activity of hsa‐miR‐4639‐5p (Figure 5e,f). Since TSA and SAHA primarily inhibit Class I, II, and IV HDACs, while SB and PB predominantly target Class I and II (Xu et al., 2007), we suspected the difference in effect might be caused by different inhibition spectrum of the inhibitors and targeting Class IV HDACs specifically may be an effective means to alter expression of hsa‐miR‐4639‐5p.

To screen for the specific class of HDACs involved in hsa‐miR‐4639‐5p regulation, a panel of HDAC inhibitors with relative specificity for different classes of HDACs were used to treat SH‐SY5Y cells. We disposed inhibitors against Class I HDACs (MS‐275), Class II HDACs (TM‐195), Class III HDACs (EX527), and Class I + IV HDACs (MGCD0103; since there are no available selective inhibitors for Class IV HDACs). Our results showed that only MGCD0103 enhanced the expression of the hsa‐miR‐4639‐5p, while MGCD0103 and MS‐275 did not change the transcriptional activity (Figure 5g,h), again suggesting Class IV HDACs might play the most important role in the regulation of hsa‐miR‐4639‐5p.

### 2.7 HDAC11 is the key histone deacetylase regulating hsa‐miR‐4639‐5p

**FIGURE 6:**
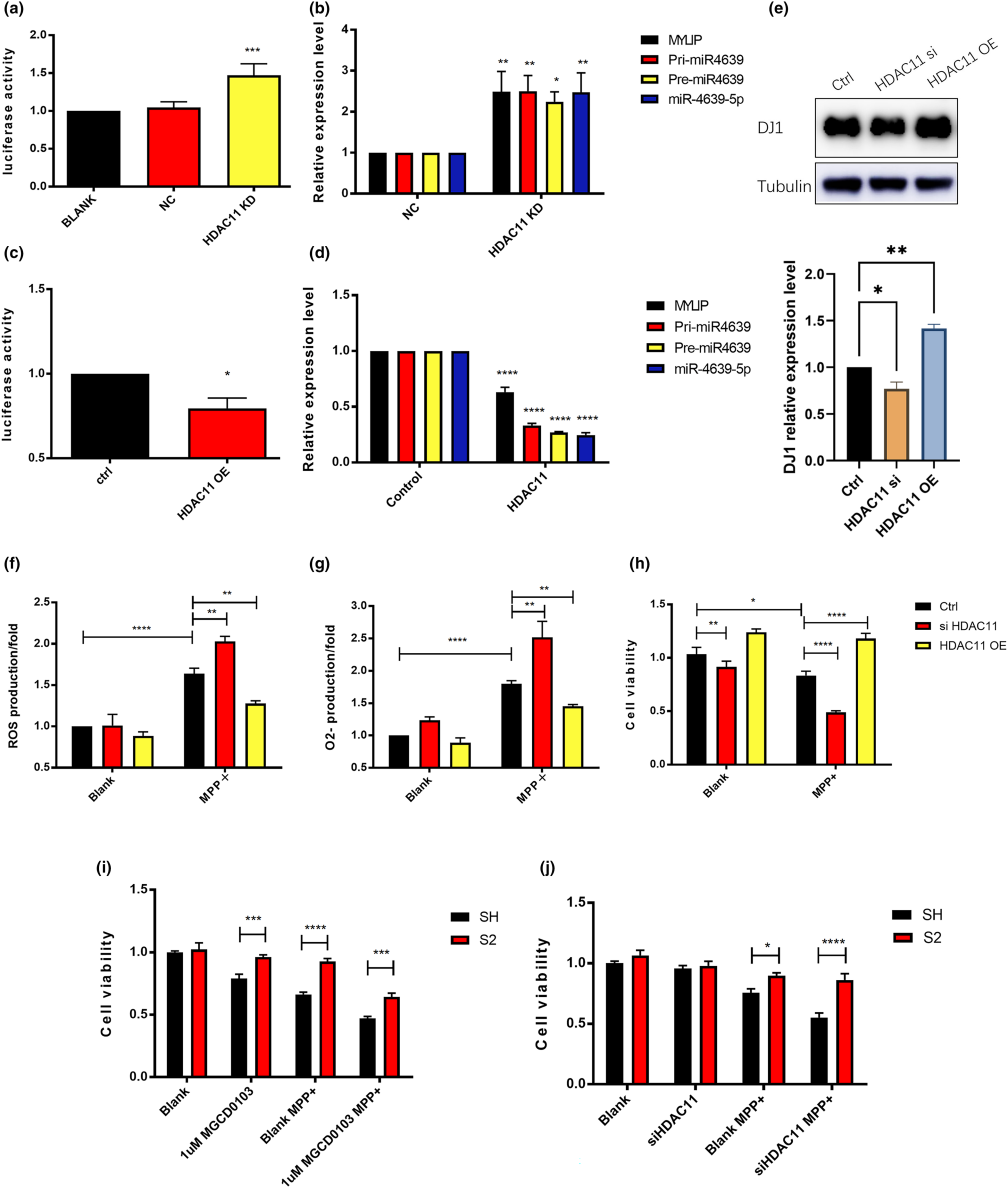
HDAC11 is the key HDAC modulating the hsa‐miR‐4639‐5p expression, the DJ‐1 protein level, and the cell antioxidative stress ability. Effects on transcriptional activity of hsa‐miR‐4639‐5p by knocking down (a) or overexpressing HDAC11 (c) examined with dual‐luciferase assay. Effects on expression level of MYLIP, pri‐, pre and mature miR4639‐5p by knocking down (b) or overexpressing HDAC11 (d) assessed with qPCR assay. (e) Effects of the knocking down or overexpressing HDAC11 on DJ‐1 protein level. The ROS production (f), O2‐ production (g), and cell viability (h) of SH‐SY5Y cells after knocking down or overexpressing HDAC11, treated with or without MPP+. (i) Cell viability of core promoter region KO cell line (S2) and control cell line with class I + IV HDACs inhibitor MGCD0103 treated and then MPP+ attack or not. (j) Cell viability of core promoter region KO cells (S2) and control cell line with HDAC11 knockdown and then MPP+ attack or not. Student unpaired t test, two‐way ANOVA with “Dunnett's multiple comparisons test”, bars are mean ± SEM. *p < 0.05, **p < 0.01, ***p < 0.001, ****p < 0.0001, compared with the ctrl/blank group.

The HDAC11 is the only member in the class IV HDAC. To investigate whether HDAC11 is the key HDAC enzyme regulating hsa‐miR‐4639‐5p expression, we knocked down HDAC11 and found that the transcriptional activity of hsa‐miR‐4639‐5p was increased. On the contrary, overexpression of HDAC11 led to a decrease in the transcriptional activity of hsa‐miR‐4639‐5p (Figure 6a,c). For control purpose, we also either knocked down or overexpressed Class I HDACs (HDAC 1, 2, 3, 8, respectively). Neither approach showed any effect on the transcriptional activity of hsa‐miR‐4639‐5p (Figure S5a,b). Furthermore, by knocking down or overexpressing HDAC11, the expression level of MYLIP, pri‐, pre‐ and mature hsa‐miR‐4639‐5p showed the same tendency in changing transcriptional activity (Figure 6b,d). Knockdown or overexpression of HDAC11 changed DJ‐1 protein level; knockdown of HDAC11 enhanced hsa‐miR‐4639‐5p expression thus downregulated the protein level of DJ‐1 (Figure 6e), while overexpression of HDAC11 reduced hsa‐miR‐4639‐5p expression and increased DJ‐1 level.

To measure the effect of HDAC11 on cell antioxidative stress activity, we assayed ROS and $O_{2}^{-}$ in SH‐SY5Y cells. By knocking down HDAC11, cells produced more ROS and $O_{2}^{-}$ and had reduced cell viability compared with untreated cells under MPP+ attack, while overexpressing HDAC11 decreased ROS and $O_{2}^{-}$ production and improved cell survival than control cells (Figure 6f–h). To ask whether the effects of HDAC11 on cell antioxidative activity were caused by its regulation on hsa‐miR‐4639‐5p, the cell line with hsa‐miR‐4639‐5p core promoter region deletion (S2) was used. Under the treatment of Class IV HDAC inhibitor MGCD0103 or MPP^+^, the control SHSY cell line showed a reduced cell viability. However, the S2 cell lines treated with Class IV HDAC inhibitor MGCD0103 or MPP^+^ S2 cells, cell viability was not affected (Figure 6i). Likewise, in normal cells with HDAC11 knockdown, MPP^+^ treatment induced a sharp decrease in cell viability, whereas MPP^+^ did not have any effect in S2 cells (Figure 6j). The results have demonstrated that HDAC11 influences the cell viability by regulating the expression of hsa‐miR‐4639‐5p.

## 3 DISCUSSION

MicroRNAs have emerged as potential biomarkers to facilitate early diagnosis of many neurodegenerative disorders (Dhawan, 2021; Gui et al., 2015). It has been suggested that the alterations of microRNAs in blood could reflect their changes in CNS under neuropathological conditions, such as neurodegenerative disorders (Cha et al., 2019; Zeng et al., 2011). For instance, in parallel with their downregulation in the brain, miR‐132 and miR‐212 were reduced in neuron‐derived plasma exosomes in AD patients (Cha et al., 2019). The distal trafficking of CNS‐derived microRNAs is thought to be mediated by the exosomes secreted by neurons and glial cells (Zhao & Zlokovic, 2017). Therefore, the abnormally changed microRNAs in blood are likely originated from the CNS (Zhao & Zlokovic, 2017).

In the present study, we purified CNS‐derived exosomes from PD plasma using two neuron surface markers (i.e., L1CAM and NCAM1) to define the source of hsa‐miR‐4639‐5p. As a result, hsa‐miR‐4639‐5p was exclusively enriched in L1CAM/NCAM1‐containing exosomes, suggesting that plasma hsa‐miR‐4639‐5p was predominately secreted by CNS neurons. We further investigated the regulatory elements of hsa‐miR‐4639‐5p and we have identified the core promoter region that plays an important role in regulating its expression. Importantly, we screened samples from PD patients and found a polymorphism in the core promoter (rs760632 G>A) that markedly enhanced hsa‐miR‐4639‐5p expression, establishing this SNP as a significant risk factor for PD. Our study has also demonstrated that hsa‐miR4639‐5p expression is regulated by HDAC11‐mediated histone acetylation.

Current study is built on our previous findings that hsa‐miR‐4639‐5p inhibited DJ‐1 expression directly by targeting the 3′UTR and suppressing the translation of *DJ‐1* mRNA resulting in neuronal cells more susceptible to MPTP‐induced oxidative insults (Chen et al., 2017). We thus hypothesized that downregulation of DJ‐1 is a novel pathogenic mechanism for sporadic PD and for which hsa‐miR‐4639‐5p plays a significant role. However, how hsa‐miR‐4639‐5p expression is regulated and by what mechanism(s) hsa‐miR‐4639‐5p expression is increased in PD are not well understood.

We reasoned that one of the mechanisms for upregulated expression of hsa‐miR‐4639‐5p could be due to the increase in hsa‐miR‐4639‐5p promoter activity. We performed screening assays and identified the core promoter of hsa‐miR‐4639‐5p located from −560 to −275 upstream the TSS of its host gene *MYLIP*. Interestingly, we found the rs760632 G>A variant that is located in the core promoter region of hsa‐miR‐4639‐5p; and this SNP likely increases PD risk by enhancing hsa‐miR‐4639‐5p expression.

Polymorphisms such as rs760632 G>A in the promoter region may influence gene expression by modulating the binding and activity of transcription factors to promoter sequences (Hua et al., 2018; Sucharov et al., 2019). Consistent with this notion, ChIP‐Seq data in the UCSC database show that the promoter binding sites for the transcription factors ELF1 and IKZF1 are near the rs760632 G>A SNP. It is plausible that the presence of rs760632 allele enhances the binding of these transcription factors to increase hsa‐miR‐4639‐5p expression. Future study will be needed to address these possibilities.

In addition to promoter polymorphisms, epigenetics changes in promoter activity such as DNA methylation/de methylation and histone acetylation/deacetylation can also influence expressions of microRNAs (Pavlou & Outeiro, 2017). Although the outcomes of such modifications are complex, DNA methylation is often associated with inhibition of gene expression, while histone acetylation is linked to transcriptional enhancement (Daskalaki et al., 2018; Tamura et al., 2012). Not surprisingly, epigenetics acts as a critical player in PD etiology and pathogenesis (Pavlou & Outeiro, 2017). Our present study has established that increased expression of hsa‐miR‐4639‐5p in PD is not mediated by DNA demethylation, but rather by histone acetylation. Our results are consistent with published literatures. For instance, in a mouse model of PD, Hsp90 expression was increased via HDAC6‐mediated deacetylation leading to enhanced chaperone‐mediated autophagy. As a result, α‐synuclein toxicity was alleviated and motor function was improved in this PD model (Du et al., 2021). We demonstrate herein that HDAC11‐mediated histone deacetylation increased the expression of hsa‐miR‐4639‐5p. Therefore, one of the implications of our current study is that interventions targeting and increasing HDAC11 activity may represent a novel therapy for PD.

It would be ideal to confirm our results in vivo. However, unlike human DJ‐1, the mouse DJ‐1 is regulated by mmu‐miR‐494 different from the miR‐4639‐5p presented in this study. It inevitably restrained the study to prove the importance of miR‐4639‐5p in a mouse model. The exact role of hsa‐miR‐4639‐5p in human PD will be studied based on organoid model in our future research. An in vivo study is also needed to further strength this discovery that plasma miR‐4639‐5p mainly originates from the CNS. Nevertheless, our current study has provided novel insights into the regulatory mechanism of hsa‐miR‐4639‐5p and DJ‐1 in the pathogenesis of PD. We provided strong evidence that the neuronal‐originated hsa‐miR‐4639‐5p may not only serve as a diagnostic marker but also a potential therapeutic target for PD.

## 4 EXPERIMENTAL PROCEDURES

### 4.1 Ethical statement

The study was approved by the Ethics Committee of Ruijin Hospital, Shanghai Jiao Tong University School of Medicine. All procedures performed in this study involving human participants were in accordance with the ethical standards of the institutional and national research committee and with the Declaration of Helsinki. Written informed consents were obtained from all participants prior to the commence of the study.

### 4.2 Antibodies and reagents

The following antibodies and reagents were used: anti‐L1CAM antibodies (Abcam, clone UJ127), normal mouse IgGs (Santa Cruz Biotechnology, sc‐2025), rabbit polyclonal anti‐PARK7/DJ‐1 antibody (Abcam, ab18257), mouse monoclonal anti‐β‐actin antibody (Sigma‐Aldrich, Clone AC‐15), horseradish peroxidase (HRP)‐conjugated goat anti‐mouse IgG, goat anti‐rabbit IgG (Jackson ImmunoResearch Laboratories). Dynabeads® Antibody Coupling Kit (Life Technologies), 5‐(and‐6)‐chloromethyl‐20,70‐dichlorodihydrofluorescein diacetate, acetylester (CM‐H2DCFDA) reagents (Invitrogen), Tetramethylrhodamine, methyl ester (TMRM; Invitrogen), Dihydroethidium (DHE) reagents (Beyotime Biotechnology), and 1‐methyl‐4‐phenylpyridinium (MPP^+^; Sigma‐Aldrich, D048).

Cell lines used in Figure 2c: 293T: human embryonic kidney cell line, SH (SH‐SY5Y): human neuroblastoma cell line, HeLa: human cervical cancer cell line, U937: human histiocytic lymphoma cell line, SW1990: human pancreatic cancer cell line, BXPC3: human pancreatic cancer cell line—epithelial source, 16H13E: human lung epithelial cell line, 95D: human lung giant cell carcinoma cell line, A549: human lung cancer cell line, SPC: human lung adenocarcinoma cell line, 1299: human lung cancer cell line, 231: human breast cancer cell line, MACF7: human breast cancer cell line, THP1: human monocyte (from Acute Monocytic Leukemia disease), U87: human glioma cell line (astrocyte), and U251: human glioma cell line (astrocyte).

### 4.3 Human subjects and samples

From March 2014 to December 2015, 358 sporadic PD patients and 407 age‐ and sex‐matched healthy controls were recruited from the department of Neurology, Ruijin Hospital. The inclusion and exclusion criteria have been described in our previous research (Chen et al., 2017). Briefly, the diagnosis of PD was established by a senior movement disorder specialist according to the diagnostic criteria for PD (Gibb & Lees, 1988). Any subject had “more than one relative with PD” was excluded. None of the subjects carried DJ‐1 L166P mutation or DJ‐1 exon 1–5 deletion, which are associated with autosomal recessive early‐onset PD (Bonifati et al., 2003), and confer reduced DJ‐1 protein level (Moore et al., 2003). All subjects underwent clinical evaluations including medical history, physical, and neurological examinations, neuropsychological assessments, and necessary laboratory tests. All the participants were of Chinese Han ancestry. Written informed consents were obtained from all participants.

Blood samples were obtained from all subjects as previously described (Elliott & Peakman, 2008). Plasma was immediately separated by centrifuging the blood at 3000 rpm for 10 min. After the lysis of erythrocytes, blood samples were centrifuged at 1223 *g* for 10 min to isolate leukocytes. DNA was extracted from leukocytes through standardized phenol/chlorine extraction method. For reservation, plasma and DNA samples were stored at −80°C.

### 4.4 Exosome isolation

Total exosomes were isolated by ultracentrifugation from plasma as previously reported (Baranyai et al., 2015; Rekker et al., 2014). CNS‐derived exosomes were isolated from the total plasma exosomes using antibody‐coated magnetic microbeads following a protocol adapted from Shi et al (Shi et al., 2014). In brief, 5 μg of anti‐L1CAM antibodies (Abcam, clone UJ127) or normal mouse IgG (Santa Cruz Biotechnology, sc‐2025) as negative control were coated onto 1 mg of M‐270 Epoxy beads using a Dynabeads® Antibody Coupling Kit (Life Technologies). One mg of antibody‐coated beads and 900 μL of PBS‐diluted total plasma exosomes were incubated at 4°C for 24 h with gentle rotation. The beads were then washed with 1 mL 0.1% bovine serum albumin (BSA)/PBS for four times and were resuspended in different buffers for subsequent experiments. For electron microscopy imaging, exosomes were eluted from the beads with 60 μL of a 1:1 mixture of 0.1% BSA/PBS and a fixing buffer (4% paraformaldehyde/5% glutaraldehyde; for protein extraction and Western blot analysis, the beads were lysed in 110 μL of 1% Triton X‐100 and 10% of a protease inhibitor cocktail in 0.1% BSA/PBS for 1 h with gentle shaking at room temperature; for RNA extraction, the beads were lysed in TRIzol LS reagent).

Two hundred ul of plasma was used for RNA isolation and expression measurement of plasma hsa‐miR‐4639‐5p. The exosome isolation method allowed us to start from 2 mL plasma and to use half of the whole exosome pellet (total exosome) and half of the entire volume of the supernatant (exosome‐free plasma) for RNA isolation. The RNA isolated from each source was dissolved in a volume of 25 μL. The amount of hsa‐miR‐4639‐5p in total exosome (exosome pellet) and in exosome‐free plasma (supernatant) was calculated by determining their threshold cycle (Ct) by real‐time quantitative (RT‐qPCR). The other half of the whole exosome pellet was used to enrich L1CAM‐containing exosomes and the total L1CAM^+^ exosome pellet (L1CAM^+^ exosome) and the entire volume of the supernatant (L1CAM^−^ exosome) were used for RNA isolation and hsa‐miR‐4639‐5p measurement. The Ct is defined as the cycle number at which the fluorescence emission exceeds a fixed threshold. Delta delta CT method was used to calculate hsa‐miR‐4639‐5p expression. The hsa‐miR‐4639‐5p in each component was normalized to the expression level of endogenous microRNA hsa‐miR‐16. Exosomes extracted from plasma samples were extracted in batches, and samples of PD patients and controls were distributed into each batch. In each batch, two reference plasma samples pooled from healthy controls were added, to eliminate batch variations.

The technique to precipitate CNS NDEs was first described in 2014, which were isolated using antibody‐coated magnetic beads against neural cell adhesion molecules L1CAM (Shi et al., 2014). Characterization of NDEs follows guidelines endorsed by the International Society for Extracellular Vesicles, which includes Nanoparticle Tracking Analysis (NTA) to determine NDEs concentration and the average diameter, Western blots for NDEs markers, and transmission EM for NDEs visualization (Shi et al., 2014). The technique was then used widely to study the changes of exocrine protein and microRNA contents derived from the CNS in neurodegenerative diseases (Jiang et al., 2020; Winston et al., 2016; Zou et al., 2020), traumatic brain injury (Peltz et al., 2020), and brain tumors (Wachowiak et al., 2018). However, recent research reported that L1CAM mainly exists in soluble forms in CSF and plasma (Norman et al., 2021), which raised the concern of the property of using L1CAM as the marker for isolating CNS NDEs. But other researchers commented the existing data in the article still cannot rule out the existence of exosome‐bound L1CAM in the form of a membrane protein. Thus, in this study, to avoid the influence of soluble L1CAM, we first isolated total plasma exosome using ultracentrifugation, and then used excess L1CAM antibody to capture CNS NDEs, and finally used NTA, Western blots, and transmission EM to prove that we captured L1CAM‐containing exosomes successfully. Except for L1CAM, NCAM1 was also used as a marker for mutual confirmation since NCAM1 was also considered as a target for immunoprecipitation of CNS NDEs due to its high and relatively specific expression in neural cells (Delpech et al., 2019; Fiandaca et al., 2015; Figure S1).

### 4.5 Transmission electron microscopy (TEM)

Isolated exosome preparations were layered onto the 300 mesh copper grids (Polysciences, Warrington), and allowed to precipitate for 1 min. The extra floating liquid at the edge of the copper mesh was absorbed by using a filter paper. Grids were then stained with 2% uranyl acetate in water and left at room temperature to dry. Imaging was performed under 80 kV using a Tecnai G2 Spirit BioTwin transmission electron microscope (FEI Company).

### 4.6 NTA

An exosome suspension aliquot (10 μL of exosome sample in 90 μL PBS) was diluted to final dilution of 1:200 in PBS. The mean diameter (nm) and concentration (particles/mL) of exosomes were determined using the Nanoparticle Tracking Analyzer ZetaView S/N 17‐310 (PARTICLE METRIX).

### 4.7 Quantitative real‐time PCR

Total RNA was extracted from plasma or exosomes with TRIzol LS reagent (Invitrogen) and from cells with TRIzol reagent (Invitrogen). cDNAs of specific genes/microRNAs were synthesized with Takara RT reagent kit according to the manufacturer's instructions. Delta delta CT method was used to calculate genes/microRNAs expression. Plasma microRNA expression results were normalized to the expression level of endogenous microRNA hsa‐miR‐16; cell microRNA expression results were normalized to the expression level of U6; MYLIP, pri‐miR‐4639, and pre‐miR‐4639 expression results were normalized to that of human actin. RT primers and qPCR primers used are listed in Data S1. All PCR products were sequenced and identified correct.

### 4.8 Western blot analysis

Equal amounts of proteins from different samples were added and separated on 10% SDS‐PAGE gels and then electrotransferred to PVDF membranes. The membranes were blocked with 5% BSA and probed with specific antibodies following the manufacturers' instructions. The blots were captured using ChemiDoc (Bio‐Rad) and quantitated using ImageLab 3.0.1 software (Bio‐Rad).

### 4.9 Promoter‐luciferase plasmids construction and luciferase assays

Primers used for the generation of 5′‐end truncated promoter fragments are described in Data S1. Fragments were digested with *KpnI* and *XhoI* and subcloned in the pGL3 basic vector (Promega). The deletion of the −560 to −275 fragment on the wild‐type 5′‐end truncated hsa‐miR‐4639 promoters were achieved with the primers described in Data S1 by overlapping PCR. Finally, the −560 to −275 fragment‐deleted promoter‐luciferase constructs were cloned in the pGL2‐basic vector (Promega). The promoter plasmid containing rs760632 A allele was amplified from the genomic DNA of PD patients with the primers used to amplify the −560 to −275 fragment. 980 μg of a reporter plasmid and 20 μg of pRL‐SV40, a Renilla luciferase vector as the internal control of firefly luciferase, were co‐transfected to SH‐SY5Y cells approximately 60% confluent in a 12‐well plate with lipofectamine 3000 (Invitrogen). For the rs760632 luciferase assay in Figure S4d, 490 μg of promoter plasmid containing rs760632 G allele and 490 μg of A allele was transfected to mimic the GA genotype. The luciferase assay was performed using a Microplate reader (Synergy Mx, Bio‐Tek) according to the manufacturer's instructions. The results presented are the relative luciferase activity generated from five independent experiments and normalized against the activity of Renilla luciferase from pRL‐SV40.

### 4.10 Deletion of specific DNA fragment by CRISPR‐Cas9 system in SH‐SY5Y cells

The pHB‐gRNA‐cas9‐Zsgreen plasmid used to generate sgRNA plasmids was obtained from HanBio company. The sgRNAs targeting specific locations were designed using the online tool CRISPR DESIGN (http://CRISPR.mit.edu). The sgRNA sequences were shown in Data S1. The sgRNAs were synthesized, annealed, and ligated to the pHB‐gRNA‐cas9‐Zsgreen plasmid that was digested with *BbsI* (New England Biolabs). Subsequently, plasmids were transformed into *E. coli* DH5α by heat shock and colonies were screened by PCR and sequencing. Positive clones were propagated followed by plasmid purification using the EndoFree Plasmid MaxiKit (Qiagen). The pHB‐gRNA‐cas9‐Zsgreen plasmids harboring corresponding sgRNAs were transfected into SH‐SY5Y cells. Twenty‐four hours after transfection, the cells expressing green fluorescence protein were sorted with flow cytometry and single cells were seeded into 96‐well plates relatively to select single cell‐derived cell lines. About two to 3 weeks after plating, single colonies were picked and proliferated. Genomic DNA was extracted from cells using Cell Genomic DNA Extraction Kit (Tiangen Biotech). The PCR products generated with specific primer sets were electrophoresed in 2% agarose gels and sequenced. Primers used for genotyping were listed in Data S1. Since the target site recognition of CRISPR‐Cas9 strictly requires the short protospacer adjacent motif (PAM), the successfully knockout cell line S2 had a piece of 439 bp promoter sequence removed, which contained the 286 bp core promoter region. Luciferase assays showed knocking out the 439 bp sequence sharply reduced the transcriptional activity, which was similar to knocking out the 286 bp core promoter region. For the S2 cell line, off‐target sequences of sgRNAs were predicted with the online tool CRISPR DESIGN (http://CRISPR.mit.edu) and 12 predicted off‐target loci were sequenced and showed no off‐target mutagenesis. Primers used for the off‐target assessment were listed in Data S1. Cells with specific promoter region deleted were cultured for the subsequent experiments.

### 4.11 Measurement of ROS, $O_{2}^{-}$, and mitochondrial membrane potential and evaluation of cell viability

Intracellular ROS or $O_{2}^{-}$ production was measured by CM‐H2DCFDA assay or DHE assay respectively. Mitochondrial membrane potential was measured with TMRM. SH‐SY5Y cells were seeded in a 12‐well plate at the number of 60,000 cell/well. Twenty‐four hours after transfecting plasmid or drug treatment, cells were treated with or without neurotoxic substance MPP^+^ (0.8 mM) for another 24 h. Cell growth medium was removed and cells were washed with pre‐warmed PBS. For ROS measurement, two μM of CM‐H2DCFDA was used to incubate with cells for 30 min at 37°C. The medium was aspirated, and cells were washed with PBS twice. The intracellular ROS was used with 488 nm laser for excitation and 530 nm emission for detection. For $O_{2}^{-}$ measurement, 10 μM of DHE was incubated with cells for 30 min at 37°C. The intracellular superoxide level of cells was measured with a flow cytometry using 488 nm laser for excitation and 610 nm emission for detection. For mitochondrial membrane potential measurement, 100 nM of TMRM was used to incubate with cells for 30 min at 37°C. The mitochondrial membrane potential was measured with a flow cytometry using 488 nm laser for excitation and 570 nm emission for detection.

For cell viability measurement, SH‐SY5Y cells were seeded in a 96‐well plate at the number of 5000/well. Twenty‐four hours after transfecting plasmid or drug treatment, cells were treated with or without MPP^+^ (0.8 mM) for another 24 h. Ten μL of CCK‐8 reagent (Beyotime Biotechnology) was added to each well. The viability of the cells was measured 2 h later at 450 nm with a microplate reader (Synergy Mx, Bio‐Tek).

### 4.12 DNA isolation, bisulfite conversion, and methylation analysis with Methyltarget^tm^ assay

Genomic DNA was isolated from plasma leukocytes through standardized phenol/chlorine extraction method. Bisulfite was converted using the EZ DNA Methylation‐Gold Kit (Zymo Research) according to the manufacturer's protocols to deaminate unmethylated cytosine to uracil; meanwhile, 5‐methyl‐cytosine was protected from deamination. Two CpG sites in the promoter of miR‐4639 were selected (Figure 4b,c). Then, for multiplex PCR amplification, 2 μL bisulfite DNA, 0.1 μM of each primer, 1 U HotStarTaq polymerase (Takara), 1× PCR reaction buffer (Takara), 0.2 mM dNTP, and 3 mM Mg^2+^ were used in a 20 μL reaction mixture. The PCR amplification program started with sample denature at 94°C for 4 min, followed by 10 cycles of the following: 94°C for 20 s, 63°C decreasing 0.5°C per cycle for 30 s, and 72°C for 60 s. Then, 25 cycles of the following: 94°C for 20 s, 65°C for 30 s, and 72°C for 60 s The PCR reaction was finalized at 72°C for 3 min. Subsequently, 1 μL of diluted PCR amplicons were used in 20 μL reaction mixture for index PCR reaction. The reaction mixture contained 0.3 μM of F primer, 0.3 μM of index primer, 1U Q5™ DNA polymerase (NEB), 1× reaction buffer (NEBQ5™), and 0.3 mM dNTP. The PCR program started at 98°C for 30 s, followed by 10 cycles of the following: 98°C for 10 s, 65°C for 30 s, and 72°C for 30 s. The reaction was finalized at 72°C for 5 min. TIANGEN Gel Extraction Kit (TIANGEN) was used to purify PCR amplicons (170–270 bp), which were then loaded onto Illumina NextSeq 500 (Illumina) and analyzed according to the manufacturer's instructions.

### 4.13 Drug treatment

DNMT inhibitors decitabine (MCE) and 5‐Azacytidine (MCE) were dissolved in DMSO at final concentration of 10 mM. To assess the effects of demethylation, SH‐SY5Y cells were grown in the presence of 3–5 μM decitabine or 5‐AzaC or DMSO (control) for 2–5 days. Culture media was changed every 24 h containing fresh DNMT inhibitors or DMSO (control). HDAC inhibitors were reconstituted in DMSO: TSA (MCE), SAHA (MCE), SB (Selleck), PB (Selleck), MS‐275 (MCE), TM‐195 (MCE), EX527 (MCE), and MGCD0103 (MCE). To assess the effects of histone acetylation, SH‐SY5Y cells were treated with 0.5 μM TSA or 5 μM SAHA or 5 mM PB or SB or DMSO (control) for 24–96 h. Culture media was changed every 24 h containing fresh pan‐HDAC inhibitors. To screen the specific class of HDACs regulating miR‐4639, SH‐SY5Y cells were treated with 0.5 μM MS‐275 or TM‐195 or EX527 or MGCD0103 or DMSO (control) for 24–96 h.

### 4.14 Chromatin immunoprecipitation assays

ChIP was performed using the SimpleChIP® Plus Sonication Chromatin IP Kit (CST, #56383) according to the manufacturer's instructions. Briefly, SH‐SY5Y cells were seeded in 15 cm dishes, treated with 0.5 μM TSA or DMSO (control) in 20 mL medium for 48 h, and cultured to reach about 90% confluency. Cells were fixed with 1% formaldehyde for 10 min and then quenched in glycine, washed, and lysed in 1 mL 1× lysis buffer + PIC. Samples were snap‐frozen and thawed on ice. The nuclei were palleted and lysed in 1 mL Nuclei lysis/sonication buffer + PIC and sonicated (Branson Digital Sonifier D250 probe) on 50% power sonicating for 1 s with 1 s interval for 90 cycles. Aliquots of cells were diluted in 1x ChIP buffer and pre‐cleared with mixed Protein G beads. Samples were then rotated at 4°C for 16 h with relevant antibodies. Antibodies bound to chromatin were incubated with Protein G magnetic beads at 4°C for 2 h and were then washed three times with low salt buffer and one time with high salt buffer. By heating at 65°C in 1× ChIP elution buffer for 30 min, chromatin was eluted from the beads; subsequently, DNA was de‐crosslinked and purified with DNA purification spin column provided in the kit. Antibodies were H3k27ac (CST #8173) diluted in 1:100 per IP, normal rabbit IgG (CST #2729) 1 μg per IP as negative control, and Histone H3 (CST #4620) 10 μL per IP as positive control. QPCR with Fast SYBR Green Master Mix (Applied Biosystems) and primers to amplify miR‐4639 promoter region (Figure 5a,b) was conducted to measure relative enrichment of the sample relative to input (% input). A gene desert negative control primer was also examined to exclude non‐specific binding. Primer sequences are listed in Data S1.

### 4.15 Statistical analysis

All data are expressed as mean ± SEM experiments of at least three independent experiments. Statistical analyses were performed using Prism6 (GraphPad Software). For column comparison, student's *t* test or one‐way ANOVA with Bonferroni's post hoc test was used. For grouped analysis, two‐way ANOVA with Dunnett's multiple comparisons test was used. All analyses were two‐tailed and were considered statistically significant when *p* values less than 0.05.

## AUTHOR CONTRIBUTIONS

JD designed and supervised the study. JD, YC, and LH wrote the manuscript. LH and YC performed experiments and analyzed data. SL was involved in the data analysis. RS generated core promoter region CRISPR‐Cas9 KO cells. HP contributed data on bioinformatics and was involved in data analysis. SC and YW recruited participants and collected blood samples.

## CONFLICT OF INTEREST STATEMENT

The authors declare that the research was conducted in the absence of any commercial or financial relationships that could be construed as a potential conflict of interest.

## Supporting information


**Figure S1.** Elevated plasma hsa‐miR‐4659‐5p in PD patients mainly exists in CNS neuron‐derived exosomes.
**Figure S2.** Long fragment downstream of the translation starting site ATG (−20~+2877) had no contribution on transcriptional activity.
**Figure S3.** Expression level of MYLIP has no effect on the DJ‐1 expression.
**Figure S4.** rs760632 G>A variation in the core promoter of hsa‐miR‐4639‐5p enhances the transcription of hsa‐miR‐4639‐5p and increases the risk of PD.
**Figure S5.** HDAC class I is not involved in the histone acetylation regulation of the hsa‐miR‐4639‐5p.
**Figure S6.** Serum hsa‐miR‐4639‐5p level in PD patients and controls.

**Data S1.** Primer list.

## Data Availability

The data that support the findings of this study are available from the corresponding author upon reasonable request. Notes: other detailed methods are available in Appendix S1.
