# Supplementary material for: Regulation of Hsa‐miR‐4639‐5p expression and its potential role in the pathogenesis of Parkinson's disease
Source: Aging Cell. 2023 Apr 26;22(6):e13840. doi: 10.1111/acel.13840 (PMC10265165; doi:10.1111/acel.13840)
Supplement: Supplementary file 1 — Figure S1. Elevated plasma hsa‐miR‐4659‐5p in PD patients mainly exists in CNS neuron‐derived exosomes. Figure S2. Long fragment downstream of the translation starting site ATG (−20~+2877) had no contribution on transcriptional activity. Figure S3. Expression level of MYLIP has no effect on the DJ‐1 expression. Figure S4. rs760632 G>A variation in the core promoter of hsa‐miR‐4639‐5p enhances the transcription of hsa‐miR‐4639‐5p and increases the risk of PD. Figure S5. HDAC class I is not involved in the histone acetylation regulation of the hsa‐miR‐4639‐5p. Figure S6. Serum hsa‐miR‐4639‐5p level in PD patients and controls. [file ACEL-22-e13840-s002.docx]

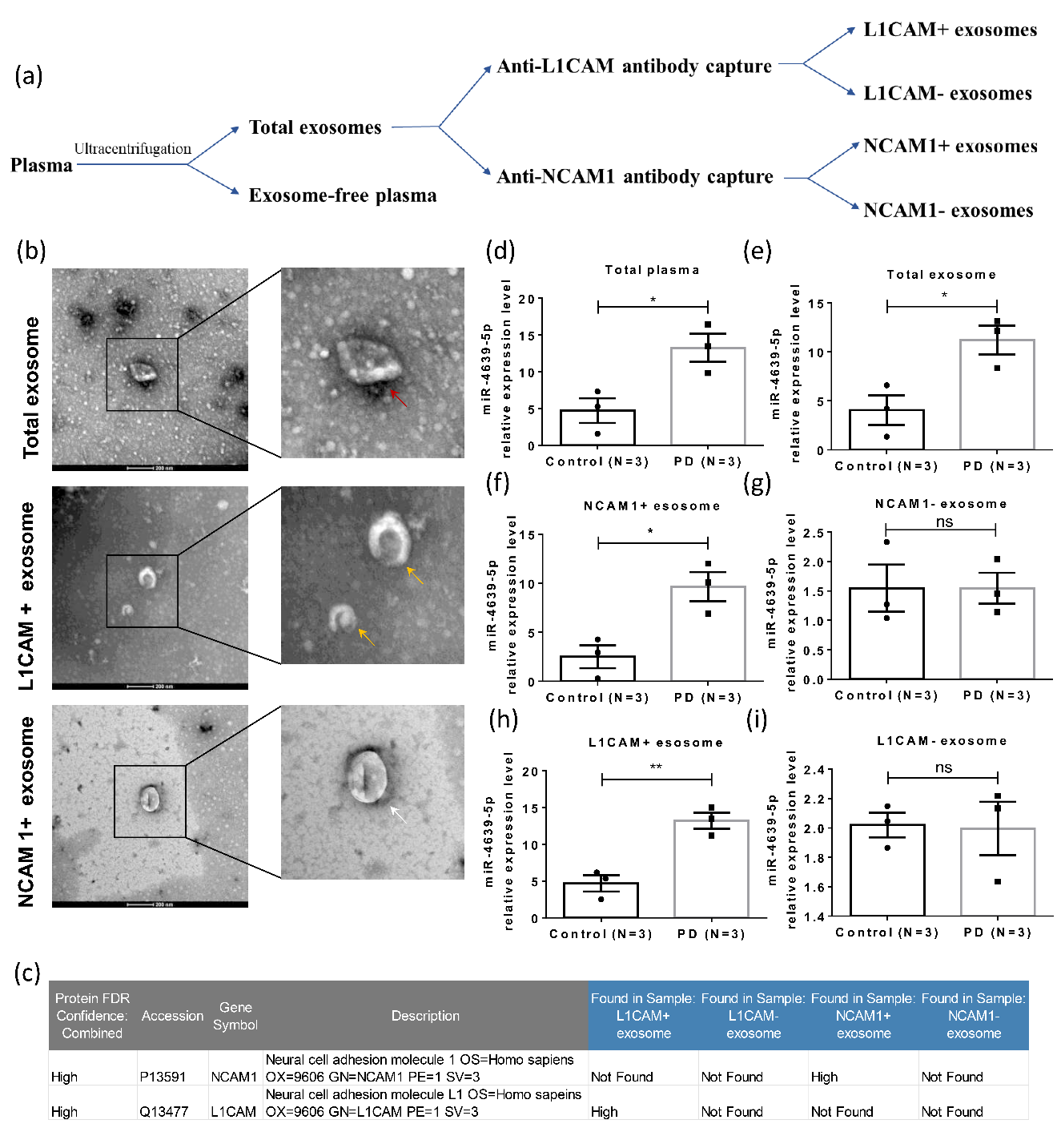


**Figure S1. Elevated plasma has-miR-4659-5p in PD patients mainly exists in CNS neuron-derived exosomes.** (a) The schematic diagram of isolating neuron-derived exosomes from plasma. (b) Electron microscopy analysis of total exosomes (indicated with red arrow), anti-L1CAM-captured plasma exosomes (yellow arrows) and anti-NCAM1-captured plasma exosomes (white arrow). Scale bar, 100 nm. (c) Mass spectrometry was performed for L1CAM (+) exosomes, L1CAM (-) exosomes, NCAM1 (+) exosomes and NCAM1 (-) exosomes. The concentration of hsa-miR-4639-5p in plasma (d), total exosomes (e), neuron-derived exosomes (f and h) and non-neuron-derived exosomes (g and i) of PD patients and controls were examined with ddPCR assay normalized to endogenous has-miR-16 expression level.


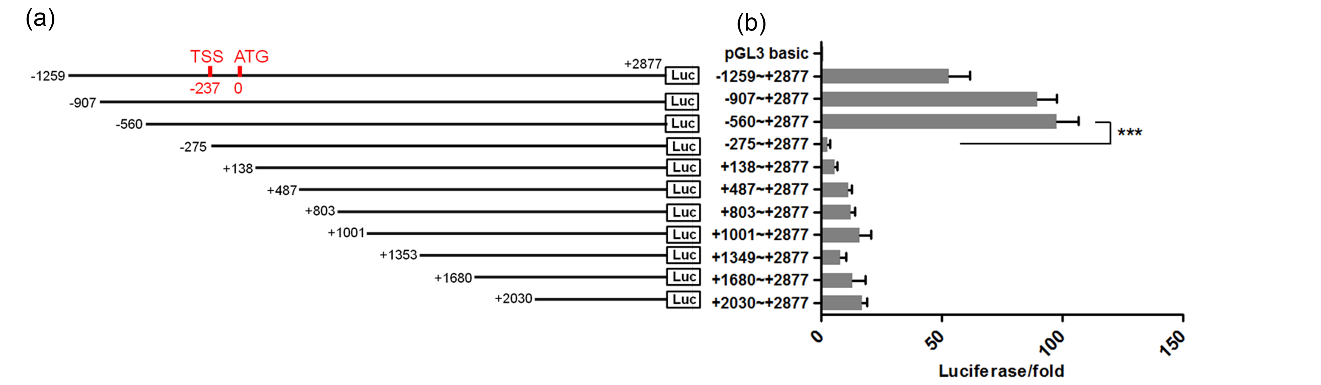


**Figure S2. The long fragment downstream of the translation starting site ATG (-20~+2877) had no contribution on transcriptional activity.** (a) Schematic representation of dual-luciferase reporter constructs containing different fragments of -1259 to +2877 across the translation starting site (ATG). (b) Dual-luciferase assay in human SH-SY5Y cells transfected with luciferase reporter vectors listed in (a). The normalized luciferase activity, as the mean+s.e.m. of at least three independent experiments is shown.

**
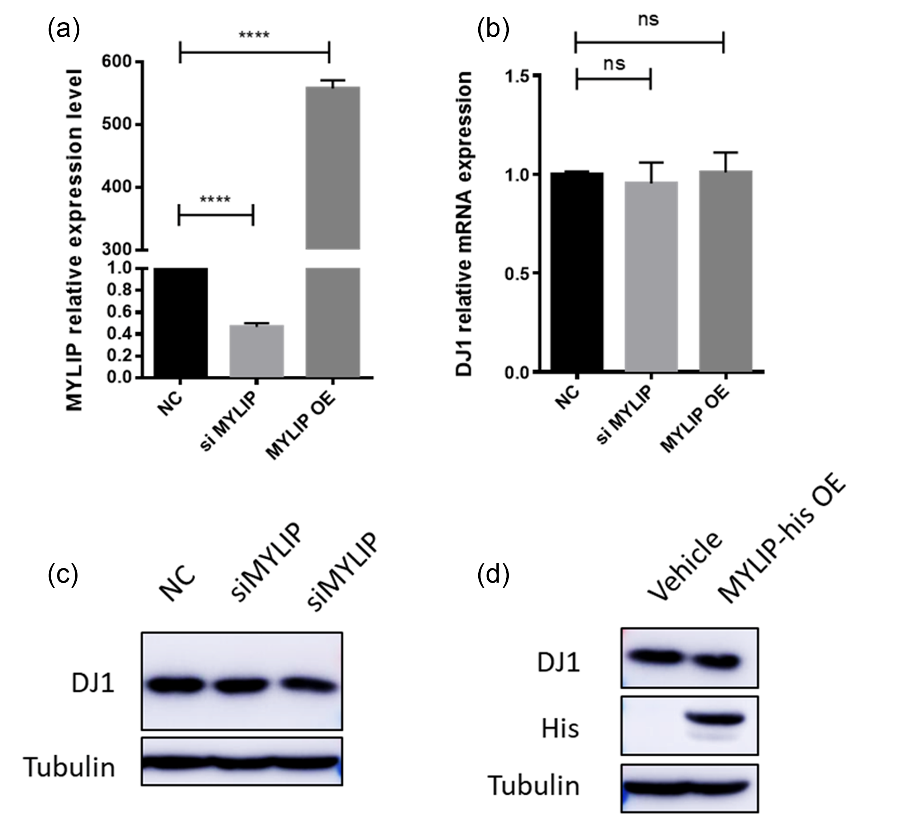
**

**Figure S3. The expression level of MYLIP has no effect on the DJ-1 expression.**

**
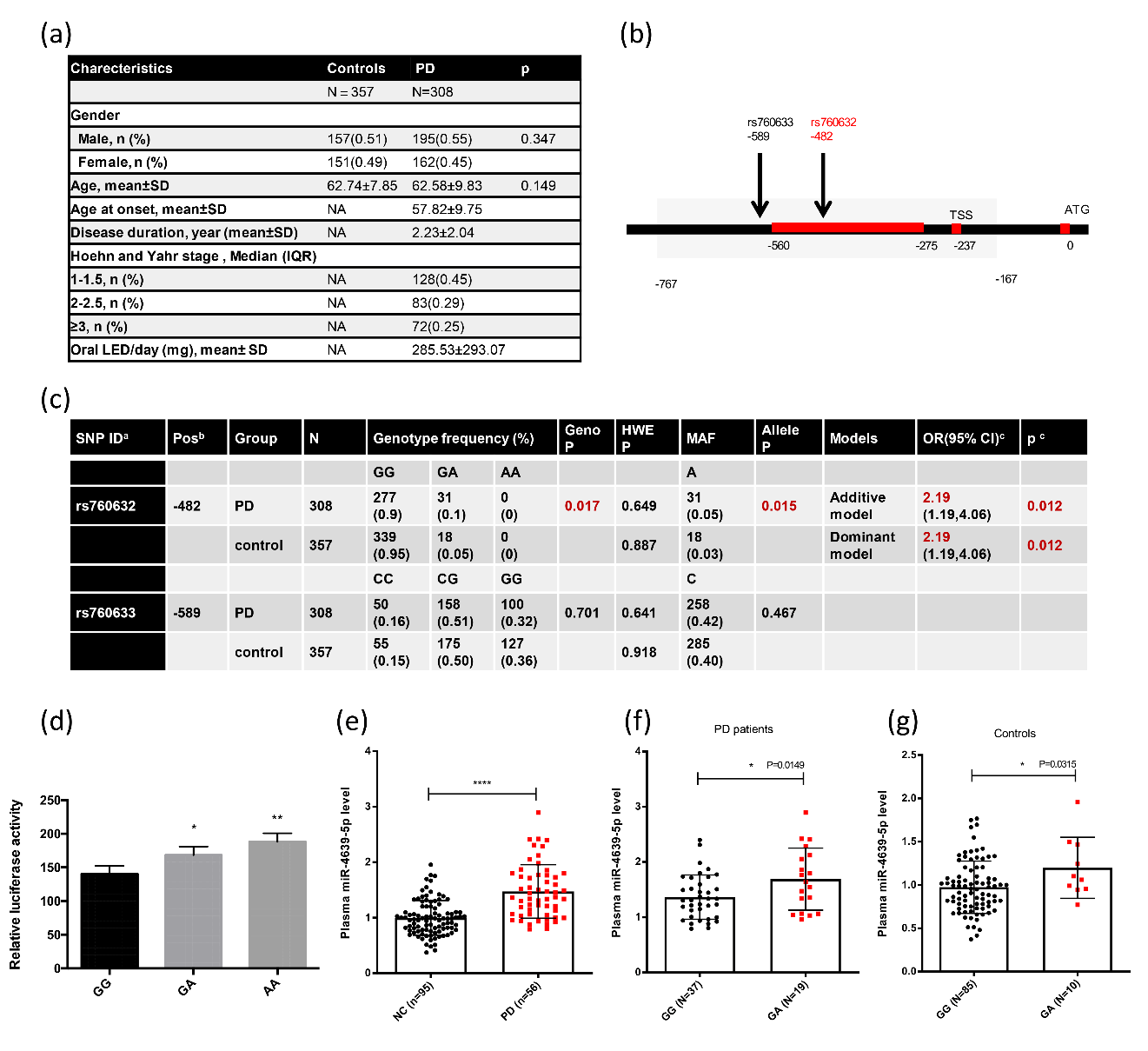
**

**Figure S4. rs760632 G>A variation in the core promoter of hsa-miR-4639-5p enhances the transcription of hsa-miR-4639-5p and increases the risk of PD.** (a) The demographic and clinical characteristics of PD patients and controls in the genotyping study. (b) The diagram of the relative locations of the core promoter region of hsa-miR-4639-5p and the SNPs identified in the study. (c) Genotype and allele distribution between PD patients and controls of the SNPs in or near hsa-miR-4639-5p promoter region. (d) Luciferase assay to explore the influences of each rs760632 genotype on the transcriptional activity of the hsa-miR-4639-5p promoter. (e) qPCR assay was used to examine the plasma hsa-miR-4639-5p level in PD patients and controls and showed PD patients had a higher plasma hsa-miR-4639-5p level than controls. (f) In PD patients and (g) controls, the persons with rs760632 GA genotype have a higher plasma hsa-miR-4639-5p level than persons with GG genotype.


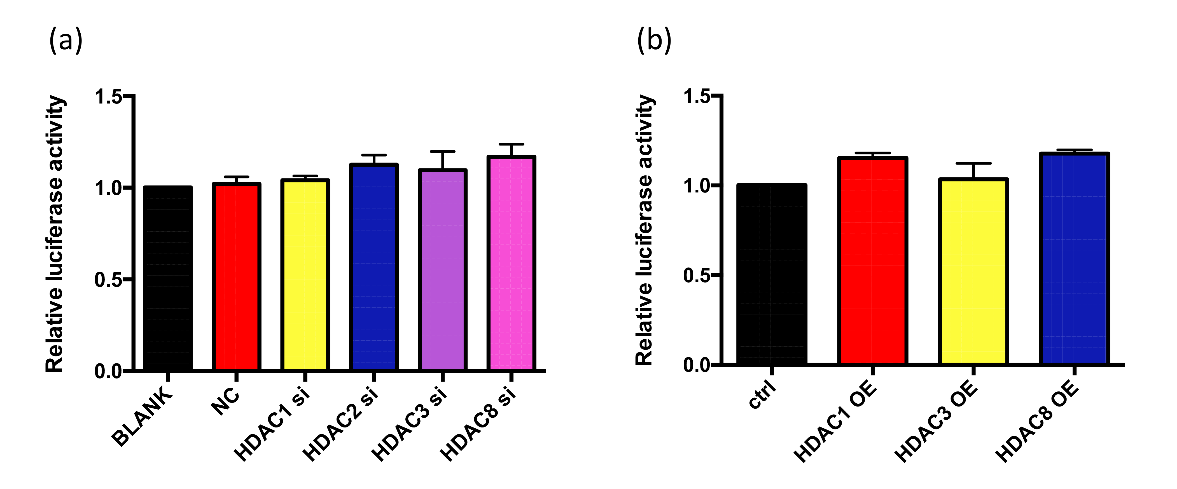


**Figure S5. HDAC class I is not involved in the histone acetylation regulation of the hsa-miR-4639-5p.**

**
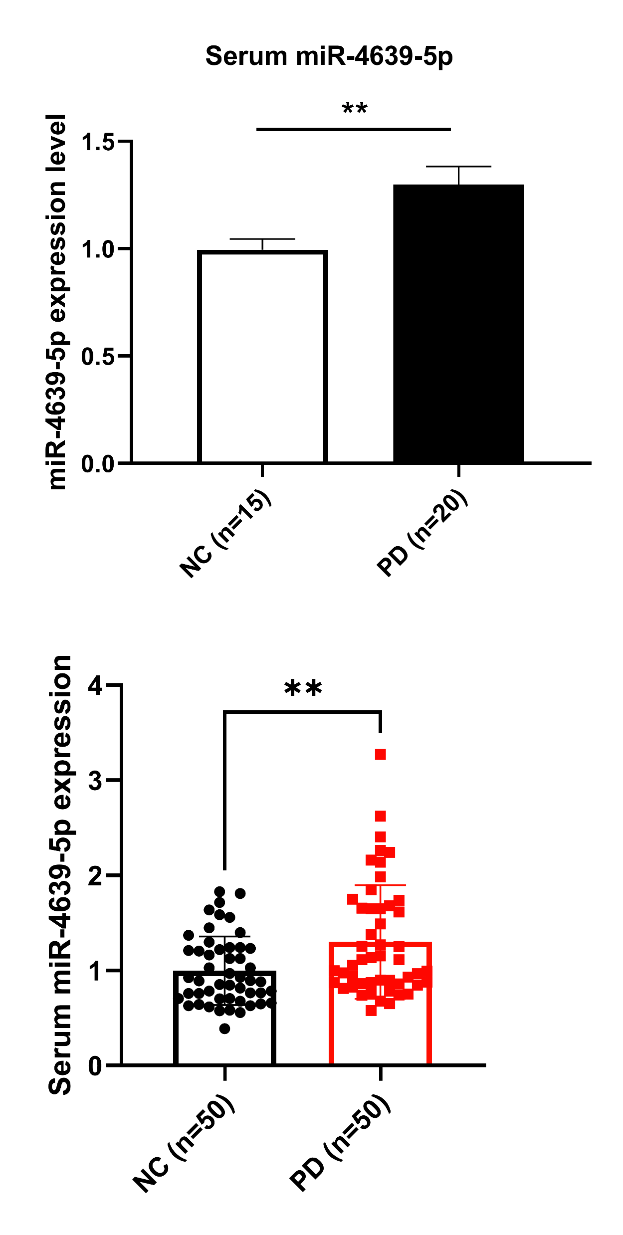
**

**Figure S6. Serum hsa-miR-4639-5p level in PD patients and controls.**
